# Expression of neuroepithelial transforming gene 1 is enhanced in oesophageal cancer and mediates an invasive tumour cell phenotype

**DOI:** 10.1186/1756-9966-32-55

**Published:** 2013-08-14

**Authors:** Conor Lahiff, Eoin Cotter, Rory Casey, Peter Doran, Graham Pidgeon, John Reynolds, Padraic MacMathuna, David Murray

**Affiliations:** 1 Gastrointestinal Unit, Mater University Hospital, Dublin 7, Ireland; 2 University College Dublin Clinical Research Centre, University College Dublin School of Medicine and Medical Science, Dublin 4, Ireland; 3 Department of Surgery, St. James’s Hospital and Trinity College Dublin, Dublin 8, Ireland

**Keywords:** Neuroepithelial transforming gene 1, NET1, Oesophageal cancer, Guanine nucleotide exchange factor, Gastrointestinal oncology

## Abstract

**Introduction:**

Neuroepithelial Transforming Gene 1 (NET1) is a well characterised oncoprotein and a proven marker of an aggressive phenotype in a number of cancers, including gastric adenocarcinoma. We aimed to investigate whether NET1 plays a functional role in oesophageal cancer (OAC) and its pre-malignant phenotype Barrett’s oesophagus.

**Methods:**

Baseline NET1 mRNA levels were determined by qPCR across a panel of six cell lines, including normal oesophageal, Barrett’s and OAC derived cells. Quantification of NET1 protein in OAC cells was performed using Western blot and immunofluorescence. NET1 expression was modulated by treating with lysophosphatidic acid (LPA) and NET1-specific siRNA. The functional effects of NET1 knockdown were assessed *in vitro* using proliferation, migration and invasion assays.

**Results:**

NET1 expression was increased in Barrett’s and in OAC-derived cells in comparison to normal oesophageal cells. The highest expression was observed in OE33 a Barrett’s-related OAC cell line. NET1 protein and mRNA expression was enhanced by LPA treatment in OAC and furthermore LPA treatment caused increased proliferation, migration and invasion in a NET1-dependent manner. NET1 knockdown resulted in reduced OAC cell proliferation and invasion.

**Conclusions:**

As found in other malignancies, NET1 expression is elevated in OAC and its pre-malignant phenotype, Barrett’s oesophagus. NET1 promotes OAC cell invasion and proliferation and it mediates LPA-induced OAC cell migration.

## Introduction

Cancer of the oesophagus consists of two major histological subtypes - squamous cell carcinoma and adenocarcinoma. These clinically, biologically and morphologically distinct cancers, display different epidemiology and mandate different clinical approaches to their management. Adenocarcinoma occurs in the lower third of the oesophagus and oesophago-gastric junction and shares much in terms of phenotype with gastric cancer. Similar to gastric cancer, intestinal metaplasia can be a prominent precursor lesion in adenocarcinoma of the oesophagus [1,2]. This condition is known as Barrett’s oesophagus. Barrett’s can represent a pre-malignant stage for oesophageal cancer and can manifest as low risk (non dysplastic) lesions or higher risk lesions showing dysplasia histologically which can be low or high grade. Oesophageal cancer (OAC) usually presents late with symptoms such as dysphagia, weight loss, substernal pain or pressure or systemic symptoms and this is reflected by poor 5 year survival figures (less than 10% for patients with advanced disease [3]).

Neuroepithelial Transforming Gene 1 (NET1) is a guanine nucleotide exchange factor (GEF) which acts via activating RhoA [4]. Rho proteins belong to the Ras superfamily of GTPases and are involved in regulating the actin cytoskeleton, signal transduction and gene transcription. These molecules bring about their downstream effects by their GTPase activity, shuttling between an inactive GDP-bound and an active GTP-bound state. This cyclical activation/inactivation brings about a conformational change with resultant downstream effects involving a wide range of cellular processes, including cell motility [5]. Rho activation occurs in response to many cellular stimuli, including lysophosphatidic acid (LPA). LPA is a bioactive phospholipid and potent signalling molecule which acts through a family of G protein coupled receptors [6]. It induces cellular proliferation through its receptors and activation of Rho. In our previous studies LPA activation of RhoA was shown to be mediated via NET1 in gastric cancer [4]. NET1 is involved in cytoskeletal organisation and cancer cell invasion [7-10]. Initially identified in a neuroepithelioma cell line, it is tumorigenic in nude mice [11]. *In vitro* studies have shown NET1 expression to drive invasion in gastric adenocarcinoma [12]. Separately it has also been shown to be functionally important in epithelial mesenchymal transition in retinal epithelial cells [13], keratinocytes [14] and during gastrulation [15]. NET1 has previously been shown to be differentially expressed and functionally important in mediating cancer cell invasion in gastric cancer [12,16] and in squamous cell skin cancer (17). It has also been shown to be prominent in a number of other cancers [17-21] and to be a marker of poor prognosis in many of these (Table 1). Our group have previously shown NET1 to be of functional importance in breast and gastric cancer [4,12,16,22]. Recognising the mounting cellular and molecular evidence for a role for NET1 in mediating gastrointestinal (GI) cancers and coupled with the phenotypic similarities recognised in the pathogenesis of gastric and oesophageal adenocarcinomas [1], we sought to investigate and fully characterise the bioactivity of NET 1 in oesophageal cancer.

**Table 1:** A summary of current data on NET1 in other human cancers

**Cancer type**	**Role of NET1**	**Reference**
Gastric adenocarcinoma	Invasion via RhoA	Leyden et al. [12]
		Murray et al. [4]
Breast cancer	Predicts late stage and poor prognosis	Gilcrease et al. [18]
	Mediates morphine-induced cell migration *in vitro*	Ecimovic et al. [22]
Glioma	Marker of invasion and aggressive disease. Poorer survival in NET1 positive	Tu et al. [20]
Hepatocellular carcinoma	Correlates with higher histological grade	Chen et al. [17]
Cervical carcinoma	Highly expressed in cervical epithelial neoplasia and in carcinoma	Wollscheid et al. [21]

## Methods

### Cell culture

Our *in vitro* oesophageal cell line model consisted of six cell lines: Het1a an SV40 immortalised normal oesophageal cell line derived from a 25 year old male; two Barrett’s cell lines QhTERT and GihTERT previously established by hTERT immortalisation (American Type Culture Collection, Virginia, USA) that represent non-dysplastic and high grade dysplastic Barrett’s epithelium respectively; and three Barrett’s related oesophageal adenocarcinoma cell lines - OE33, OE19 and JH-EsoAd1. OE33 was established from an adenocarcinoma of the lower esophagus of a 73-year-old female patient and is pathological stage IIA and poorly differentiated. OE19 is a pathological stage III moderately differentiated adenocarcinoma of gastric cardia/oesophageal gastric junction in a 72-year-old male patient. JH-EsoAD1 is from a patient with Barrett’s associated adenocarcinoma [23]. AGS is a gastric cancer cell line from a 54 year old female and represents a moderate to poorly differentiated adenocarcinoma. SW480 is from a locally invasive (Duke’s stage B) colon adenocarcinoma. QhTERT, GihTERT, OE33, OE19, Jh-EsoAd1, AGS and SW480 cells were cultured in RPMI 1640 medium containing 10% fetal calf serum, 2 mM Glutamine and penicillin/streptomycin. Cells were cultured in T-75 flasks maintained at 37°C in a humidified atmosphere of 5% CO_2_. Het1a required a supporting layer composed of extracellular matrix proteins for subculture. Flasks were coated with 0.01 mg/ml bovine serum albumin, 0.01 mg/ml fibronectin and 0.03 mg/ml bovine type I collagen and were incubated overnight at 37°C in 5% CO_2._ Het1a was cultured in BEBM medium containing BPE 0.4%, insulin 0.5 ml, hydrocortisone 0.5 ml, gentamicin/amphotericin 0.5 ml, retinoic acid 0.5 ml, transferring 0.5 ml, triiodothyronine 0.5 ml, epinephrine 0.5 ml and hEGF 0.5 ml (Lonza Clonetics, Walkersville, USA). Flasks were maintained at 37°C in a humidified atmosphere of 5% CO_2_.

### RNA extraction and qPCR

RNA extraction was carried out using TRIzol™ reagent (Sigma Aldrich, Ireland) under standard conditions. Quantitative PCR was carried out by the SyBr Green method using the Rotor-Gene™ 3000A Real Time Thermal Cycler and the Rotor-Gene™ 6 software package. Specifically designed primers for NET-1 were purchased from Qiagen (Crawley, West Sussex, UK) and GAPDH was used as an endogenous control.

### Western blot

Following LPA stimulation or siRNA treatment, cells were lysed and total protein was analysed by immublot using SC-50392 (Santa Cruz, United States) NET1 specific rabbit IgG monoclonal antibody.

### Immuno-fluorescence

2 × 10^4^ cells were seeded into 8 well chamber slides, treated with either NET-1 specific siRNA or scramble siRNA and incubated at 37°C for 24 hours with 5% CO_2_. Immuno-fluorescence was measured using SC-81333 (Santa Cruz, United States) NET1 specific mouse IgG monoclonal antibody and a FITC labelled secondary antibody.

### Optimisation of LPA treatment by dose/response

In order to determine optimal treatment conditions for LPA in OE33 and het1a cell lines a dose/response experiment was performed. Cells were treated with 1, 5, 10 and 20 μl LPA and. NET1 mRNA expression was quantified by qPCR and protein expression was examined by Western blot.

### Gene knockdown by siRNA

Two siRNA duplexes were designed and synthesised for silencing NET1 (Qiagen Inc. CA, USA). The duplexes were termed: NET1-1 (sense, 5′- GGA GGA UGC UAU AUU GAU A-3′; antisense, 5′- UAU CAA UAU AGC AUC CUC C-3′) and NET1-2 (sense, 5′- GGU GUG GAU UGA UUG GAA A- 3′; antisense, 5′ UUU CCA AUC AAU CCA CAC C-3′). A chemically synthesized non-silencing siRNA duplex (sense, 5′-UUC UCC GAA CGU GUC ACG U-3′; antisense, 5′-ACG UGA CAC GUU CGG AGA A-3′) that had no known homology with any mammalian gene was used to control for non-specific silencing events. 4 × 10^5^ OE33 cells were added to each well of a 6-well plate containing 2 ml growth media and were incubated under the standard conditions of 37°C and 5% CO_2_ in a humid incubator for 24 hr. After 24 hrs the siRNA-containing culture medium was aspirated and 1.9 ml of new medium was added to each well. 1 μl (0.3 μg, 10nM), 5 μl (1.5 μg, 50nM), 17 μl (5 μg, 170nM) and 25 μl (7.5 μg, 250nM) siRNA were added to serum-free RPMI medium and then diluted appropriately in serum-containing medium as per manufacturer’s instructions. Each specific oligonucleotide (NET1-1 and NET1-2) was examined individually and together in the same solution. NET1 mRNA expression was quantified by qPCR and protein expression was examined by Western blot and immunofluorescence.

### Proliferation assay

20 μl of MTS reagent was added to each well of a 96 well plate containing 2 × 10^4^ cells. Treatments were as follows; 10nM scramble siRNA (control), 10nM NET1-1 siRNA, 10nM scramble siRNA + 5 μM LPA and 10nM NET1-1 + 5 μM LPA. After transfection with siRNA, cells were incubated for 24 hours. MTS was then added and the plate was incubated for 2 hours at 37°C and 5% CO_2_ and absorbance at 492 nm was read using a microplate reader.

### Migration assay

Wound healing migration assays were performed using plastic well inserts (Ibidi, Germany) in 24 well plates. 8 × 10^4^ cells were seeded to each side of a plastic insert inside each well. The following day 10nM NET1-1 siRNA was added with 10nM scramble siRNA acting as a control. Cells were incubated under standard conditions for 24 hours to achieve knockdown of NET1. Inserts were then carefully removed from each well and cells were fed with regular growth medium without siRNA. Wells for LPA treatment were treated with 5 μM in medium. Cells were observed until they had migrated but not long enough to allow full closure of the gap created by removal of the insert (3 hours). Cells were then fixed using 1:1 methanol acetone and stained with crystal violet. Each well was then photographed at 3 hours and measurements were taken for each condition at three points along the gap between mono-layers of cells. All treatment conditions were carried out in triplicate and averages were calculated and recorded as distance in number of pixels across the gap. Comparisons were made between the scramble siRNA and NET1 knockdown wells. Analysis calculated average migration distances using Image J software (http://rsb.info.nih.gov/ij/).

### In vitro invasion assay

Biocoat Matrigel (BD Biosciences, United Kingdom) invasion chambers were used to investigate and compare the effect of NET1 downregulation on the *in vitro* invasion of OE33 cells. 1 × 10^5^ cells were seeded to the upper chamber in serum-free medium. Culture medium containing 20% FBS was added to the outer chambers which acted as a chemo-attractant for the cells. The plates were then incubated for 24 hr in a 5% CO_2_ humidified 37°C incubator. Following incubation, the cells which had invaded the membrane were fixed and stained. The membrane was then removed and mounted on a slide for microscopic assessment. Invasive cells were visualised at 40X magnification and the number of cells in five random fields were counted and an average calculated for each condition.

### Statistics

All experiments were carried out in triplicate unless otherwise stated in results section. Quantitative PCR analysis was by delta Ct method and GAPDH was used to normalise the data. Bivariate statistical analysis was carried out using the student’s *t*-test with the level of statistical significance taken as p < 0.05.

## Results

### NET1 Expression is upregulated in oesophageal cancer cells

Relative NET1 mRNA expression across all six cell lines is shown in Table 2. Het1a (normal) cell line set at an arbitrary reference value of 1. There is a marked higher level of expression in the OE33 cell line. Because of this high NET1 level we chose this cell line for further experiments to characterise the role of NET1 in oesophageal cancer. Looking at other *in vitro* GI cancer models (Additional file 1: Figure S1), the OE33 cell line had greater NET1 mRNA expression compared to gastric (AGS) and colorectal (SW480) adenocarcinoma models.

**Table 2:** NET-1 mRNA expression in Barrett’s oesophagus and oesophageal cancer cell lines relative to het1a (normal) oesophageal cell line

**Cell line**	**Description**	**Mean NET1 expression**	**Standard deviation**
Het1a	Normal oesophagus	1.0	0
QhTERT	Non-dysplastic Barretts epithelium	54.8	65.5
GihTERT	High grade dysplastic Barretts epithelium	2.8	2.5
JH-EsoAd1	C	2.8	2.5
OE19	OAC	61.5	30.3
OE33	Stage IIa, poorly differentiated OAC	180.4	178.4

Specific cell lines are as identified in methods section.

### NET1 MRNA expression is modulated by targeted siRNA and LPA

**Figure 1:**
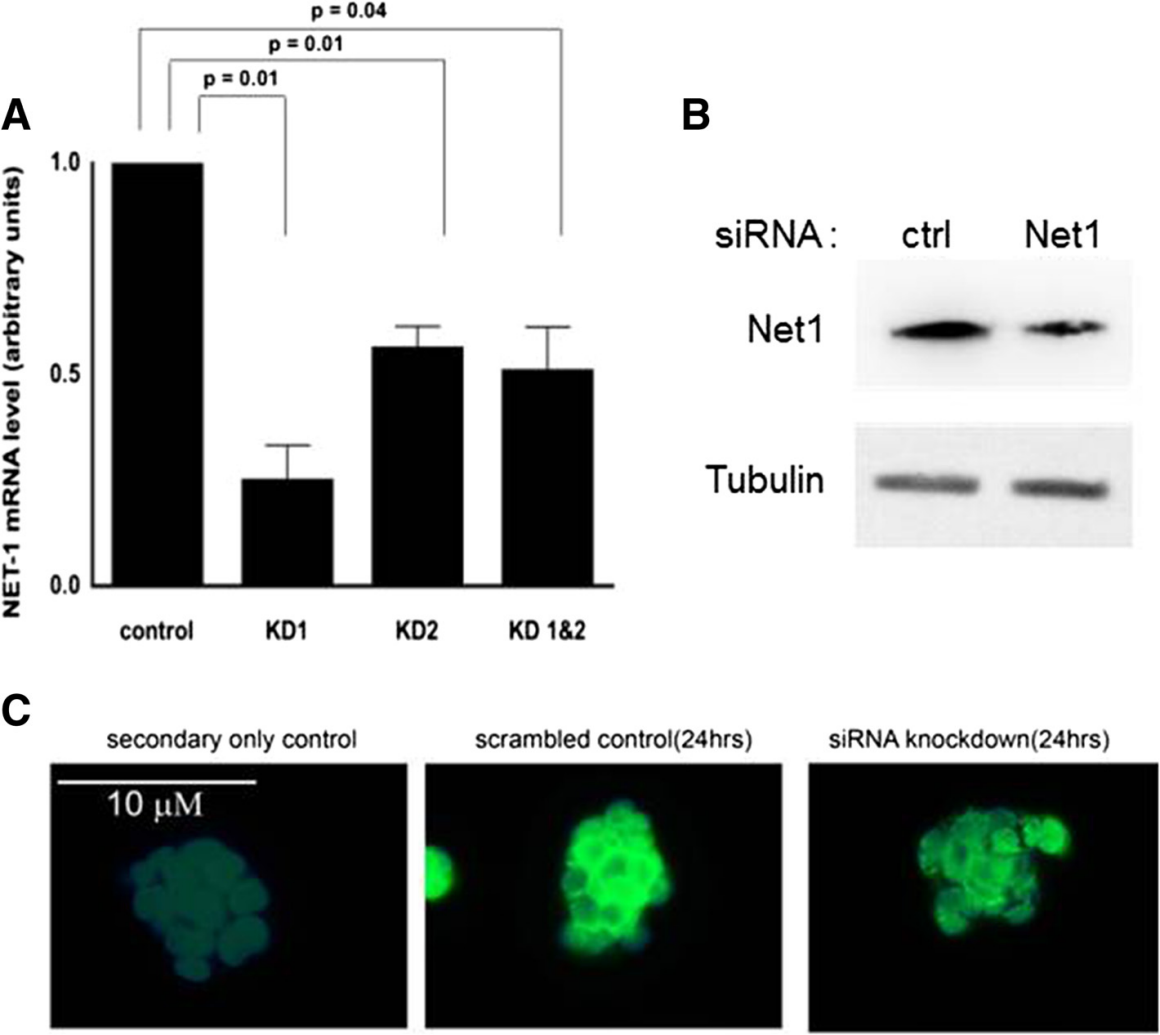
**NET1 expression following knockdown by siRNA in OE33 cells. A)** NET1 mRNA expression after gene knockdown with NET1-specific siRNA oligonucleotide 1 (KD1), NET1 siRNA oligonucleotide (KD2) and both siRNA in combination (KD 1&2). **B)** Western blot showing NET1 protein expression in OE33 cells after gene knockdown, using tubulin expression as a control. Reduced expression was seen in NET1 knockdown compared to control. **C)** Immunofluorescence images from OE33 cells after siRNA NET1 gene knockdown. Reduced fluorescence was observed for NET1 knockdown compared to (scrambled) control siRNA at 24 hours incubation. Secondary antibody control image is included for reference.

Optimal NET1 gene knockdown conditions were determined by dose–response and time-course transfections in OE33 cells. The most effective knockdown (76%) was observed at 10nM for 24 hours using NET1 duplex 1, as shown in Figure 1A (0.24 vs. control, p = 0.01). Similar effects on NET1 protein expression were shown by Western blot and immunofluorescence (Figure 1B and C).

**Figure 2:**
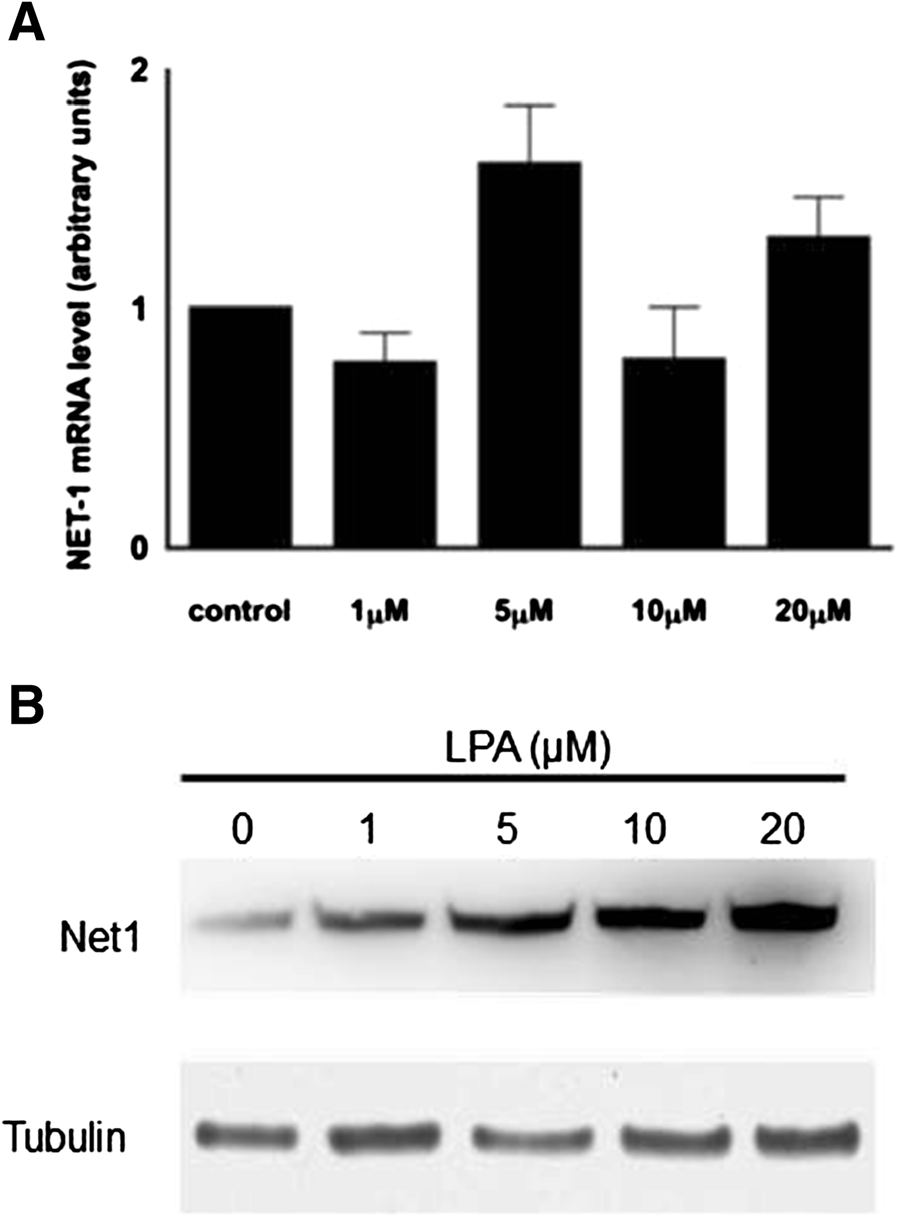
**NET1 expression following stimulation with LPA in OE33 cells. A)** Effect of LPA stimulation on NET1 mRNA expression in OE33 cells. The most pronounced effect was seen at 5 μM where a 1.6 fold rise was observed (p = 0.13). **B)** NET1 protein expression in OE33 cells after stimulation with LPA. Tubulin was used as a housekeeper.

Maximum LPA effect (1.6 fold rise in NET1 mRNA, p = 0.13) was seen at a treatment concentration of 5 μM for 4 hours, as shown in Figure 2A. Consistent with this, LPA treatment was shown to result in elevated Net1 protein levels (Figure 2B).

### NET1 Knockdown reduces OAC cell proliferation

**Figure 3:**
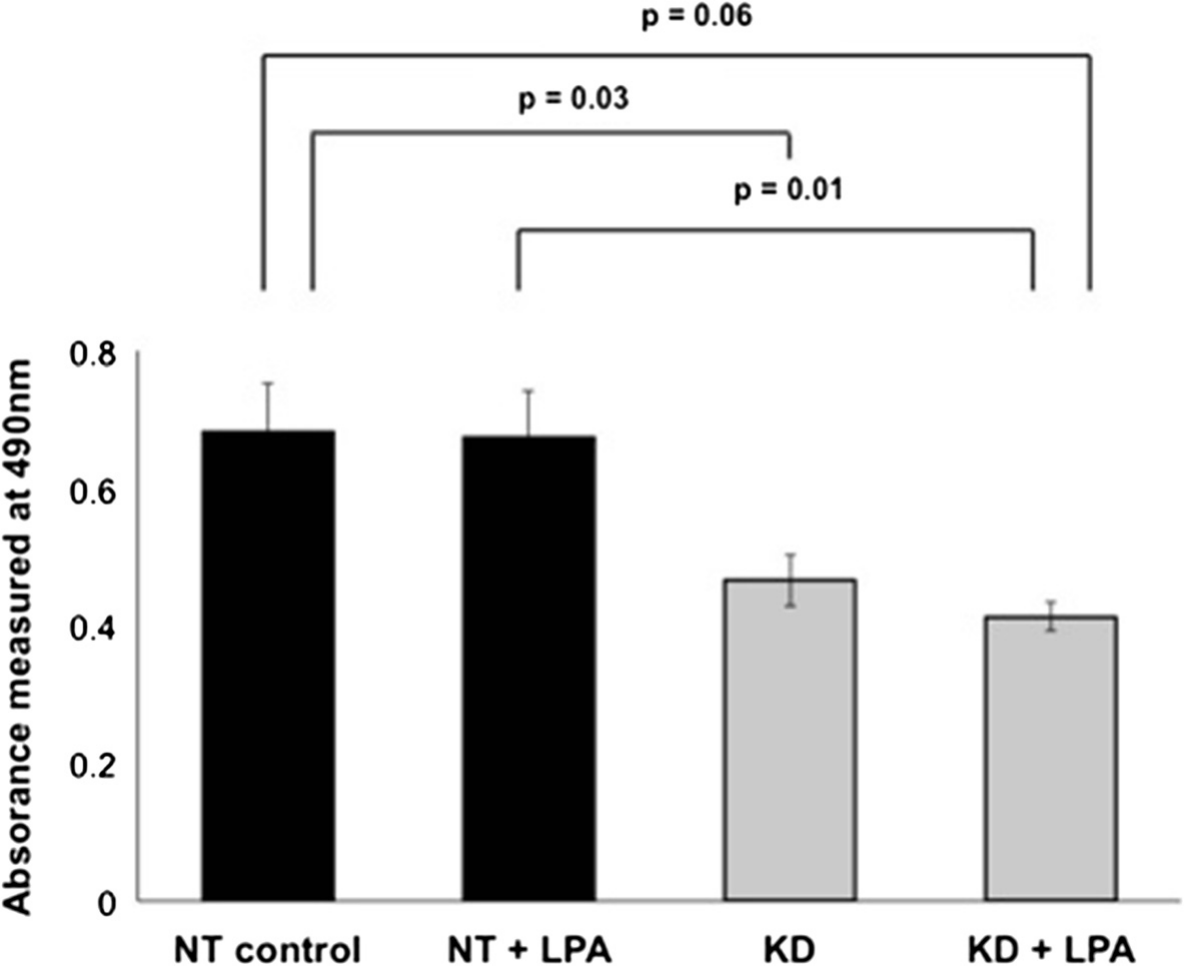
**OE33 cell proliferation measured after NET1 knockdown (KD) and 5 μM LPA stimulation compared with control (scramble siRNA) cells.** Statistically significant differences are shown in bold.

NET1 gene knockdown reduced OE33 cell proliferation by 32% (mean absorbance 0.46 versus 0.68, p = 0.03) in comparison to scramble siRNA control (Figure 3). Treatment with LPA had no significant effect on OAC cell proliferation. NET1 knockdown cells treated with LPA showed significantly reduced proliferation (39% reduction, p = 0.01) compared to control cells treated with LPA under the same conditions.

### NET1 Mediates LPA induced migration in OAC cells

**Figure 4:**
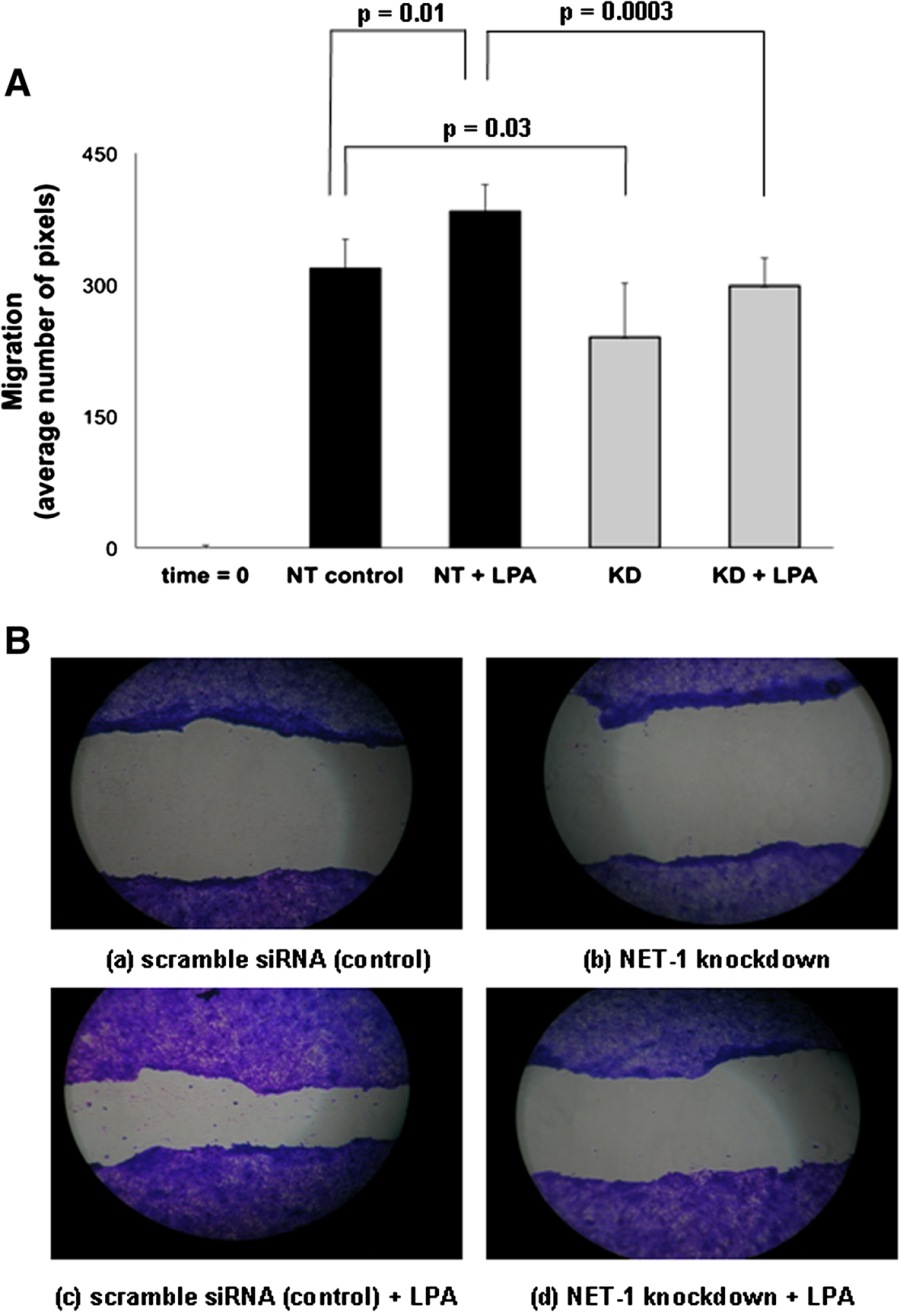
**Trans-well migration of OE33 cells after NET1 gene knockdown (KD), 5μM LPA stimulation (NT+LPA) and both conditions combined (KD+LPA). A)** Migration across a gap is graphed by average number of pixels. Non-targeting siRNA (NT control) treated cells acted as a sham control for gene knockdown and time=0 is included as a reference. Statistically significant differences are shown in bold. **B)** Light microcopy images (10× magnification) of trans-well migration assay.

Figure 4 illustrates the effects of LPA treatment and NET1 knockdown on OAC cell migration, using gap width at time 0 as a reference. A higher level of migration was observed in LPA treated cells compared to non-targeting (NT) siRNA (control) cells (383.3 mean pixels versus 318.1 or 20% increase in migration, p = 0.01). NET1 gene knockdown (KD) resulted in 25% reduction in migration (240 mean pixels versus 318.1, p = 0.03). NET1 knockdown cells treated with LPA had a 22% reduction in migration in comparison with control (NT + LPA), (298.5 versus 383.3 mean pixels, p = 0.0003).

### NET1 Promotes trans-membrane invasion in OAC cells

**Figure 5:**
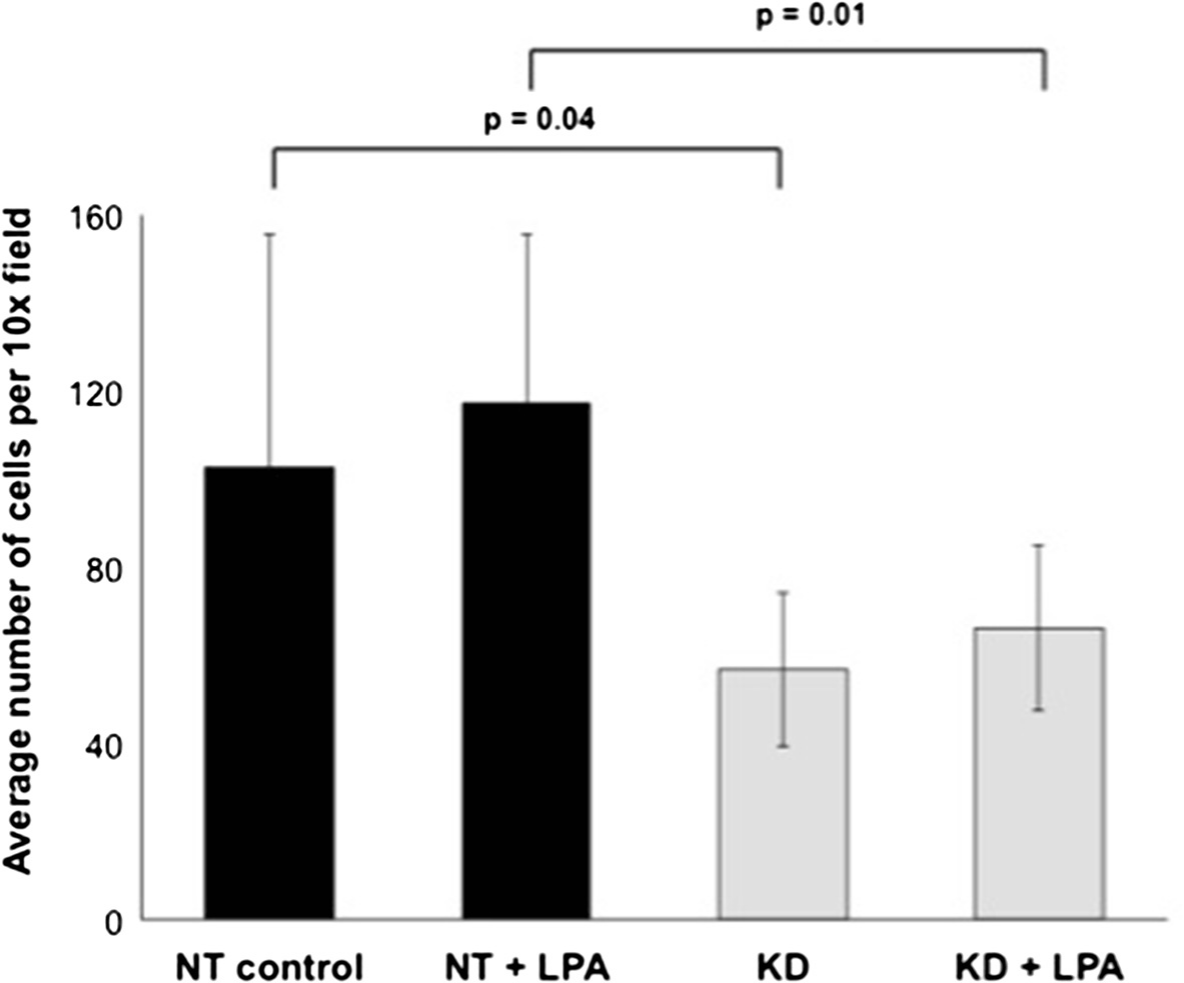
**Trans-membrane invasion of OE33 cells after NET1 knockdown (KD) and 5 μM LPA stimulation (control + LPA) over 24 hours compared with control (NT/scramble siRNA).** The final column represents both conditions combined (KD + LPA). Statistically significant differences are shown in bold.

NET1 knockdown cells were 45% less invasive at 24 hours than control cells, as shown in Figure 5 (56.8 versus 102.6 mean cells per high power field, p = 0.04). Invasion was increased by 78% in control cells after 5 μM LPA stimulation compared with NET1 knockdown cells (117.1 vs 66.1 mean cells per high power field, p = 0.01).

## Discussion

The biological events in OAC carcinogenesis and metastasis are poorly understood. NET1 has been shown to be functionally important as a mediator of invasion and metastasis in gastric adenocarcinoma [12,16] and is prognostically significant in other epithelial cancers [18,20]. We have demonstrated very high levels of NET1 expression in OAC and this strengthens our central hypothesis that this well characterised oncoprotein may be an important player in the molecular events leading to neoplastic progression in Barrett’s and OAC. Analysis of baseline NET1 expression levels in our *in vitro* oesophageal model showed a progressive rise in expression from normal oesophagus to Barrett’s to Barrett’s related OAC. The higher expression of NET1 in OE33 OAC cells compared with the other two OAC cell lines may be a reflection of the poor level of differentiation these cells represent, and it has been shown elsewhere that NET1 is seen at high levels in the later metastatic stages of other cancers [17,20]. In a recent study (Lahiff et al 2013, under review British Journal of Cancer; Lahiff, et al. Gut 2012; 61: (Suppl 2) A255 (abstract); and Lahiff et al. Gastroenterology 2012; 142:5 (Suppl 1) S-531 (abstract)].) we have analysed the levels of NET1 mRNA in OAC tumor tissue. We showed that type I (Siewert classification) oesophago-gastric junction (OGJ) adenocarcinomas expressed significantly higher levels of NET1, with lowest expression in type III and intermediate levels in type II (p = 0.01). In patients with gastric and OGJ type III tumours, NET1 positive patients were more likely have advanced stage cancer (p = 0.03), had a higher number of transmural cancers (p = 0.006) and had a significantly higher median number of positive lymph nodes (p = 0.03). In this subgroup, NET1 was associated with worse median overall (23 versus 15 months, p = 0.02) and disease free (36% versus 11%, p = 0.02) survival.

In the current study, we investigated the role of NET1 in OAC by modulating its expression and investigating the effect on cell function. LPA stimulates invasion and migration in OE33 cells. We have previously shown that LPA, a phospholipid which acts through G protein coupled receptors and is known to activate RhoA, promotes gastric cancer cell invasion via NET1 [4]. In this current study we have shown that not only does LPA drive NET1 expression in OAC but that the functional effects of LPA stimulation in these cells are NET1 dependent. Although not explored in the current study, our ongoing efforts will define whether LPA drives RhoA activation in OAC cells as it does in gastric cancer cells. The mechanism by which LPA induces transcription of NET1 in OAC cells remains to be elucidated. We also previously reported LPA to drive the expression of NET1 mRNA in gastric cancer cells [4]. Likewise, we previous showed [16] that stimulation of gastric cancer cells with LPA resulted in the differential expression of over 2000 genes. Further work will elucidate the mechanism via which LPA induces NET1 mRNA transcription in OAC cells.

The results of the functional *in vitro* experiments presented here are broadly consistent across proliferation, migration and trans-membrane invasion assays. NET1 knockdown significantly reduced OE33 cancer cell proliferation, migration and invasion. LPA, a recognised mitogen, had no effect on proliferation in these OAC cells. However, when we examine the effect of LPA on scramble siRNA control cells compared with its effect after NET1 knockdown there was significant differences in proliferation, migration and invasion. While these results suggest the effect of LPA in promoting proliferation, migration and invasion in OAC may be NET1 dependent this needs to be qualified by the fact that in control cells at baseline we only observed a significant effect after LPA treatments in the migration assay. Furthermore, although not performed in this study, it would also be valuable to monitor the effect of NET1 overexpression in OAC cells and efforts, aimed at performing these analyses are currently ongoing.

Epithelial Mesenchymal Transition (EMT) plays a key role in the metastasis of epithelial cancers through the involvement of various intracellular signalling pathways [24-26]. Loss of E-Cadherin is associated with EMT and tumour invasion [27] and has been linked functionally to NET1 and TGFβ [14]. Oesophageal cancer frequently exhibits loss of E cadherin and TGFβ receptors [28]. Interestingly RhoA, which our group have previously shown to be regulated by NET1 in gastric cancer [4], has also been shown to activate TGFβ [29]. Furthermore, we have previously shown NET1 expression to be required for the expression of TGFβi, a key member of the TGF signalling pathway [16]. TGFβ is known to induce NET1 expression and in turn RhoA activation and reorganisation of the cytoskeletal via the Smad3 transcription factor [13]. The putative role of NET1 in epithelial mesenchymal transition via TGF-β [13,14,19,30] and the significance of this concept in OAC, coupled with the data presented here, strengthen the hypothesis that NET1 plays an important role in the tumour biology of oesophageal adenocarcinoma.

## Conclusions

The data presented from this study demonstrates that NET1, a recognised pro-invasive oncoprotein associated with aggressive gastrointestinal and non-gastrointestinal cancers is highly expressed and functionally active in OAC. In aggregate our data provides strong evidence that NET1 is biologically active in OAC and may be an important factor in promoting an aggressive tumour cell phenotype.

## Abbreviations

NET1: Neuroepithelial transforming gene 1; OAC: Oesophageal cancer; GI: Gastrointestinal; GEF: Guanine nucleotide exchange factor; LPA: Lysophosphatidic acid; EMT: Epithelial mesenchymal transition.

## Competing interests

The authors declare that they have no competing interests.

## Authors’ contributions

CL: study concept and design, experimental work and acquisition of data, drafting of the manuscript, analysis and interpretation of data, critical revision of the manuscript for important intellectual content. EC, RC, GP: experimental work and acquisition of data, interpretation of data, critical revision of the manuscript for important intellectual content of the manuscript. PD, JR: analysis and interpretation of data, drafting of the manuscript critical revision of the manuscript for important intellectual content of the manuscript. PMM: study concept and design, analysis and interpretation of data, critical revision of the manuscript for important intellectual content of the manuscript. DM: study concept and design, experimental work and acquisition of data, critical revision of the manuscript for important intellectual content of the manuscript. All authors read and approved the final manuscript.

## Funding source

The Mater Foundation.

## Supplementary Material

Additional file 1: Figure S1 NET1 mRNA expression in other *in vitro* GI cancer models. OE33 cells line had highest expression of NET1 mRNA expression compared to gastric (AGS) and colorectal (SW480) adenocarcinoma models. Click here for file
