# Supplementary figures and images for: Expression of neuroepithelial transforming gene 1 is enhanced in oesophageal cancer and mediates an invasive tumour cell phenotype
Source: J Exp Clin Cancer Res. 2013 Aug 14;32(1):55. doi: 10.1186/1756-9966-32-55 (PMC3751529; doi:10.1186/1756-9966-32-55)

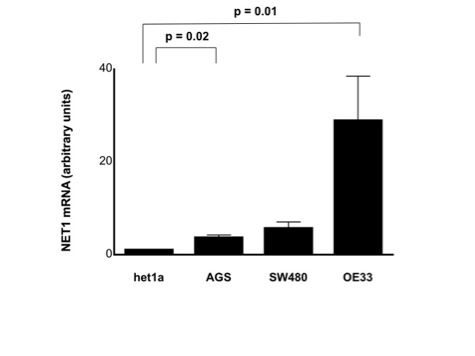

Supplement: Additional file 1: Figure S1 — NET1 mRNA expression in other in vitro GI cancer models. OE33 cells line had highest expression of NET1 mRNA expression compared to gastric (AGS) and colorectal (SW480) adenocarcinoma models. [file 1756-9966-32-55-S1.jpeg]
